# Continuous separation of bacterial cells from large debris using a spiral microfluidic device

**DOI:** 10.1063/5.0159254

**Published:** 2023-08-09

**Authors:** Ayomikun Esan, Frédérique Vanholsbeeck, Simon Swift, Cushla M. McGoverin

**Affiliations:** 1 Department of Physics, University of Auckland, Auckland, New Zealand; 2 The Dodd-Walls Centre for Photonic and Quantum Technologies, Auckland, New Zealand; 3 Department of Molecular Medicine and Pathology, University of Auckland, Auckland, New Zealand

## Abstract

With the global increase in food exchange, rapid identification and enumeration of bacteria has become crucial for protecting consumers from bacterial contamination. Efficient analysis requires the separation of target particles (e.g., bacterial cells) from food and/or sampling matrices to prevent matrix interference with the detection and analysis of target cells. However, studies on the separation of bacteria-sized particles and defined particles, such as bacterial cells, from heterogeneous debris, such as meat swab suspensions, are limited. In this study, we explore the use of passive-based inertial microfluidics to separate bacterial cells from debris, such as fascia, muscle tissues, and cotton fibers, extracted from ground meat and meat swabs—a novel approach demonstrated for the first time. Our objective is to evaluate the recovery efficiency of bacterial cells from large debris obtained from ground meat and meat swab suspensions using a spiral microfluidic device. In this study, we establish the optimal flow rates and Dean number for continuous bacterial cell and debris separation and a methodology to determine the percentage of debris removed from the sample suspension. Our findings demonstrate an average recovery efficiency of ∼80% for bacterial cells separated from debris in meat swab suspensions, while the average recovery efficiency from ground beef suspensions was ∼70%. Furthermore, approximately 50% of the debris in the ground meat suspension were separated from bacterial cells.

## I. INTRODUCTION

In the field of microbiological analysis, significant strides have been made in the development of rapid techniques for the enumeration, identification, and detection of microorganisms. However, there remains a critical need for effective methods that can efficiently separate and concentrate microorganisms from food matrices.^1^ Such processes hold immense potential to increase the speed and sensitivity of microbial detection. Dealing with debris from food and sampling matrices in microbiological analysis can pose significant challenges. The presence of debris in the initial suspension can obscure colonies during optical detection.^2^ While several detection systems have demonstrated the capability to provide prompt results within a matter of hours, their efficacy can be limited when dealing with microorganisms present in low abundance, particularly pathogenic bacteria. Thus, the development of innovative and efficient approaches for the separation and concentration of microorganisms from food samples is of utmost importance in advancing the field of microbiological analysis and ensuring food safety.

Size-based membrane filtration is one of the most popular methods for separating target particles in food sample suspensions. This process uses a porous filter to remove particles above a certain size in suspension.^3–5^ This separation technique has the drawback of clogging the filter pores with large debris, resulting in slow processing times.^4^ Centrifugation is another method used to separate target particles in food suspension. A major limitation of this method is the potential for mechanical damage to the sample particulates due to the shear forces generated during the process.^6^ Due to these limitations of membrane filtration and centrifugation, research on the application of microfluidic devices for continuous particle/cell separation has increased over the years.^5,7–14^

Microfluidics have been explored for the separation of particles in sample suspensions due to advantages including reduced sample volume, faster processing time, and low cost.^11^ Microfluidic-based separation technologies can be classified into two categories: active and passive. Active separation methods use an external force field to separate the cell, which may result in complex experimental systems.^9^ The development of passive separation techniques that do not require an external force to separate particles or cells has received much attention in recent years. Using passive separation techniques, particles/cells are separated based on intrinsic properties such as size and shape.^9,15^ The passive separation methods that have been developed include pinched flow fractionation,^16–20^ deterministic lateral displacement,^21–32^ and inertial microfluidics.^33–36^ Inertial microfluidic-based passive devices have been reported to achieve high separation efficiency at relatively high flow rates with fewer fabrication steps than active devices.^9,10,37^ However, there are some limitations to passive microfluidic devices. For example, devices based on deterministic lateral displacement using walls and posts can become clogged with larger particles and restrict essential fluid flow.^37^ In addition, passive microfluidic devices operating based on pinch flow separation will not be effective for separation experiments that involve small particles (less than 1 μm in diameter) because the inertial lift force experienced by the particles is insignificant. The application of viscoelastic microfluidics for cell separation has also been reported. For instance, Zhang *et al.*^38^ conducted a study demonstrating the separation of *E. coli* clusters from single cells in a straight microchannel using elastic forces to achieve effective cell separation. In a recent study by the same research group, they successfully demonstrated the separation of different bacterial species using viscoelastic flows in a straight microchannel.^39^ However, it is important to note that viscoelastic microfluidics necessitates the utilization of specialized viscoelastic fluids, which often have complex formulations, requiring meticulous control of parameters such as shear rates, concentration, and relaxation times.

Inertial microfluidics offers a relatively straightforward separation process compared to the other passive separation methods described earlier. Inertial microfluidics rely on the inherent hydrodynamic forces generated by fluid flow in microchannels, such as inertial lift forces, Dean drag, and secondary flow effects.^10^ Passive separation in inertial microfluidics is accomplished through inertial lateral migration, in which particles or cells are focused at specific equilibrium positions in the cross section of a microchannel using fluid hydrodynamic forces.^10^ The particle/cell size and the velocity profile of the background flow are used in this separation technique to achieve particle or cell separation. The channel geometry can be used to define a critical separation diameter, which causes particles/cells with diameters greater than the critical diameter to focus near the channel wall at an equilibrium position.^45^ Di Carlo *et al.*^37^ were among the first to validate the use of inertial microfluidics for particle size separation by continuously separating spherical particles ranging from 2 to 17 μm. Based on particle sizes and the focusing criterion $a_{\;p}/D_{H}\geq 0.07$, it was demonstrated that particles are focused into different equilibrium positions in a microchannel. A loss of focus was observed when $a_{\;p}/D_{H}\leq 0.07$ and at Dean number $D_{e}\geq 20$. The inertial focusing of non-spherical microparticles in a straight microchannel has also been reported.^46^ Incorporation of flow-focusing devices into inertial microfluidics enables the confinement of particles/cells into a narrow horizontal flow before undergoing inertial effects. Nieuwstadt *et al*.^47^ applied inertial microfluidics in a flow-focusing microfluidic device with two outlets to achieve the sized-based separation of microspheres. A separation efficiency of 69% was achieved for the 3 μm target particle. In their device, particles were lost to the side channel due to a change from ideal Poiseuille flow to a focused flow in the main channel. When using inertial microfluidics in combination with flow-focusing the loss of target particles must be considered. Further, Clime *et al*.^45^ combined hydrodynamic focusing and inertial lateral migration in a microfluidic device with a rectangular cross section to study the separation of a Gram-positive rod bacterium of dimensions 0.5 μm in diameter and 1–2 μm in length, from debris extracted from ground beef. Over 50% ground beef debris removal, as determined by optical turbidity measurements, was reported, and a 20% loss in bacteria recovery. The 20% loss of bacteria was attributed to the lateral migration of debris into two equilibrium positions, which extracts some bacteria from the central stream into the lateral sheath flow.

The development of spiral or curved microfluidic devices that can focus particulates above a critical diameter into a single equilibrium position for improved recovery efficiency has been studied (Table I). Bhagat *et al.*^13^ applied a spiral microfluidic device to achieve sized-based separation of micro-particles (1.9 and 7.32 μm) with a separation efficiency of around 90%. Kuntaegowdanahalli *et al.*^35^ applied a five-loop spiral microchannel to achieve the continuous separation of 10, 15, and 20 μm polystyrene particles with a separation efficiency of 90%. Nivedita and Papautsky^12^ achieved 95% separation efficiency of erythrocytes (red blood cells are disks ∼7 μm diameter with a thickness varying between 1 and 2.5 μm) from leukocytes (white blood cells are more spherical cells with diameter varying between 10 and 20 μm) from a diluted sample of blood. The use of spiral microfluidic devices for the separation of circulating tumor cells (CTCs) from red blood cells (RBCs) and leukocytes with a recovery efficiency of over 85% has been reported by Hou *et al.*^40^ Warkiani *et al*.^41^ reported the use of a spiral microfluidic device to study yeast cells (3–5 μm) separation from Chinese Hamster Cells (CHO) cells (10–20 μm) with a 95% separation efficiency. Spiral microfluidic devices for the separation of non-motile sperm cells with a recovery efficiency of 81% were reported by Son *et al.*^42^ A recent study by Nepal *et al.*^43^ reported a 90% sperm cell (oval-shaped head (3–5 μm length and 2–3 μm width), a mid-piece (7–8 μm), and a tail (45 μm)) recovery efficiency from red blood cells and white blood cells using a spiral microchannel with a rectangular cross section. In their study, they found that by increasing the channel area to achieve high flow throughout, they were able to increase the percentage of red blood cells removal from sperm cells. Lee and Yao^5^ reported the use of a spiral microfluidic device for the study of the separation of *Cosmarium* microalgae (circle shape with a diameter ranging from 20 to 80 μm) and *Chlorella vulgaris* microalgae (spherical shape with a diameter ranging from 2 to 10 μm diameter) with a separation efficiency between 60% and 80%. Schaap *et al.*^48^ applied spiral microchannels to the sorting of algal cells by shape and size. A separation efficiency of over 77% was achieved and it was discovered that the shape of algae can have an impact on their respective equilibrium position in the microchannel. A recent application of spiral microchannel by Lee *et al.*^44^ demonstrated the possibility of separating *E. coli* bacteria from 2.29 and 4.70 μm micro-particles with an extraction yield of 98.01%. However, this study did not investigate different bacterial species and how variations in bacterial concentration can impact recovery efficiency.

**TABLE I.** An overview of separation efficiencies achieved with spiral microfluidic devices for a variety of cell and particle separations.^a^

Application	Sample properties	Separation efficiency (%)	Reference
Particle separation	Microparticles (1.9 and 7.32 *μ*m)	90	Bhagat *et al*.^13^
Particle separation	Polystyrene particles (10, 15, and 20 *μ*m)	90	Kuntaegowdanahalli *et al*.^35^
Blood cells separation	Erythrocytes (∼7 *μ*m) from leukocytes (10–20 *μ*m)	95	Nivedita and Papautsky^12^
Tumor cells separation	CTCs (∼10–20 *μ*m) from RBC (∼8 *μ*m) and leukocytes	85	Hou *et al.*^40^
Yeast cells separation	Yeast cells (3–5 *μ*m) from CHO (10–20 *μ*m)	95	Warkiani *et al*.^41^
Sperm cells separation	Sperm cells (head length: 4.79 *μ*m) from RBC (∼9 *μ*m)	81	Son *et al*.^42^
Sperm cells separation	Sperm cells from RBC (∼9 *μ*m) and WBC (∼12 *μ*m)	90	Nepal *et al*.^43^
Microalgae separation	*Cosmarium* (20–80 *μ*m) from *Chlorella vulgaris* (2–10 *μ*m)	60–80	Lee and Yao^5^
Bacteria separation	*E. coli* from micro-particles (2.29 and 4.70 *μ*m)	98	Lee *et al*.^44^

^a^ CTCs—circulating tumor cells, RBC—red blood cell, WBC—white blood cell and CHO—Chinese Hamster cells.

Inertial microfluidics has been shown to effectively separate cells and particles based on size, and to a lesser extent, shape. However, there is limited research on separating particles in the smaller size range typical of bacteria, as well as studies specifically focused on separating defined particles (e.g., bacterial cells) from heterogeneous debris found in a meat swab. This separation is crucial for optically based enumeration methods, where interference from meat debris poses challenges (e.g., obstruction, overlapping signal).

In this study, we explore the application of inertial microfluidics for the separation of bacterial cells from debris extracted from ground meat and meat swabs. A spiral microchannel is developed to separate particles greater than 5 μm in diameter from the other particulates. The 5 μm threshold was chosen as even though bacteria exhibit a diverse size range, bacterial cells typically range from 0.4 and 2 μm in diameter and 0.5 and 5 μm in length.^49^ By selecting a critical diameter of 5 μm, our aim is to effectively separate the majority of commonly encountered bacterial species. In our design, we leverage the phenomenon of Dean drag force to manipulate the motion of bacterial cells while also harnessing inertial lift forces to focus large meat debris into equilibrium positions.

The novelty of our work lies in the utilization of inertial microfluidics for the specific application of separating bacterial cells from a mixture of debris found in meat swabs and ground meat suspension. Meat swabs and ground meat suspensions in addition to the bacterial cells of interest contain particulates, such as cotton fibers, muscle tissues, and fascia. Our methodology aims to achieve efficient and reliable separation, enabling more accurate and interference-free optical bacteria enumeration methods.

## II. THEORY AND PRINCIPLE

The phenomenon of inertial migration is the movement of particles randomly distributed across a microchannel to specific equilibrium positioning.^9^ In a laminar Poiseuille flow with a parabolic velocity profile, particles will achieve lateral equilibrium positions based on the interaction of two lift forces—shear gradient-induced and wall-induced. In a microchannel, the shear gradient lift force is driven by the parabolic velocity curvature of Poiseuille fluid, pulling particles from the microchannel center toward the walls. However, as the particles move toward the channel walls, their rotational wake is disturbed by the wall-induced lift force that pushes them away from the wall.^10,35,50,51^ The inertial lift force varies with the Reynolds number and a dimensionless lift coefficient $C_{L}$. The lift coefficient is a function of the channel Reynolds number $R_{e}$ (unitless) and particle position $x_{c}$ within the transverse section of the channel. The net inertial lift force is given as follows:^35,50^

$$F_{L}=\frac{\mu_{F}^{2}}{\rho_{F}}R_{\;p}^{2}C_{L}(R_{e},x_{c}),$$ (1)

where $\mu_{F}$ is the dynamic fluid viscosity, $\rho_{F}$ is the fluid density, $R_{\;p}$ is the particle Reynolds number (unitless), and $C_{L}$ is the lift coefficient. The channel Reynolds number can be expressed as^52^

$$R_{e}=\frac{U_{F}D_{H}\varrho_{F}}{\mu_{F}}.$$ (2)

While the particle Reynolds number ( $R_{\;p}$) is defined as follows:

$$R_{\;p}=\frac{U_{F}a_{\;p}^{2}\varrho_{F}}{\mu_{F}D_{H}},$$ (3)

where $U_{F}$ represents the fluid-flow velocity and $a_{\;p}$ represents the particle diameter. $D_{H}$ represents the hydraulic diameter of the channel and can be calculated by using the channel height ( H) and the width ( W),

$$D_{H}=\frac{2WH}{\left(W+H\right)}.$$ (4)

By balancing the net inertial lift in Eq. (1) and the Stokes drag— $F_{S}=6\pi \mu a_{\;p}U_{\;p}$—the lateral migration velocity of particles, $U_{\;p}$, can be expressed as^9^

$$U_{\;p}=\frac{F_{L}}{3\pi \mu_{F}a_{\;p}}=\frac{U_{F}^{2}a_{\;p}^{3}\rho_{F}}{3\pi \mu_{F}D_{H}^{2}}C_{L}.$$ (5)

To achieve particle inertial migration in a straight microchannel, several parameters need to be controlled including the particle diameter ( $a_{\;p}$), the hydraulic diameter of the channel ( $D_{H}$), the fluid velocity ( $U_{F}$), and an aspect ratio of the channel H/W and the optimum channel length ( $L_{c}$). The optimum channel length ( $L_{c}$) required for particles to achieve inertial equilibrium in a straight microchannel can be expressed as^10^

$$L_{c}=\frac{\pi \mu_{F}H^{2}}{\rho_{F}U_{F}a_{\;p}^{2}C_{L}}.$$ (6)

**FIG. 1.**
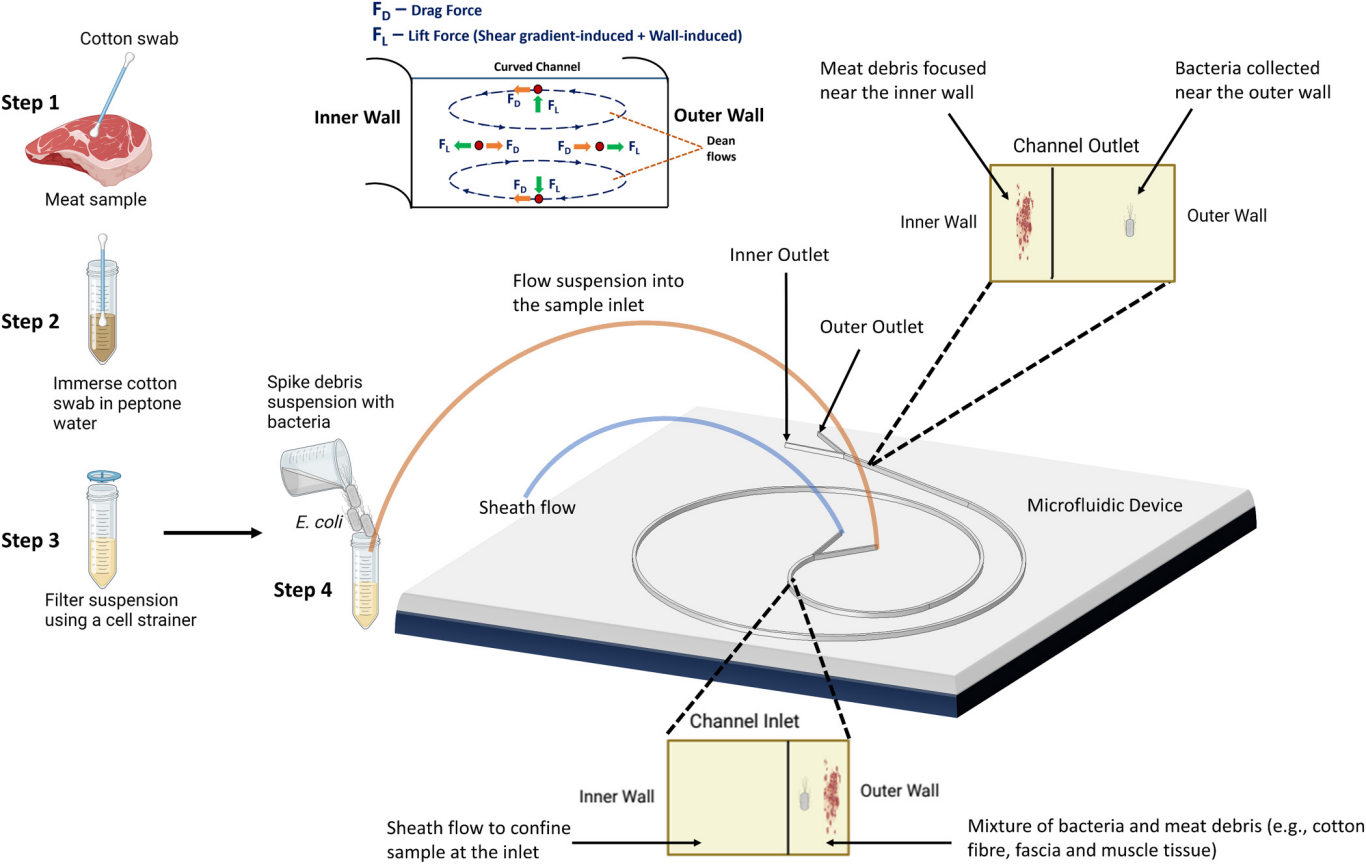
An overview of the separation method and device. The device comprises a sheath flow and sample inlet, as well as two outlets. The separation mechanism relies on the utilization of lift forces and Dean drag to facilitate the inertial migration of particles, which satisfy the focusing criterion ( $a_{\;p}/H\geq 0.07$). In a curved microchannel, the generation of a secondary flow gives rise to the formation of two counter-rotating streams known as the Dean vortices. The particles distributed within the curved microchannel experience the combined effects of the net inertial lift force ( $F_{L}$) and the Dean drag force ( $F_{D}$), which determines the particle equilibrium position based on particle size. Near the outer wall of the microchannel, both $F_{L}$ and $F_{D}$ act in the same direction, resulting in particles following the path of the Dean vortices irrespective of their size. Near the inner wall, the net lift force and Dean drag force exert opposing effects on the particles, leading to a dynamic equilibrium. “Created with BioRender.com.”

In a curved or spiral microchannel, particles are subjected to the inertial lift force (shear-induced and wall-induced) and a Dean drag force. The Dean drag force occurs in curved channels and is caused by the secondary flow patterns generated by the channel curvature. When fluid flows through a curved channel, it experiences a centrifugal effect, resulting in the formation of secondary flows that circulate perpendicular to the main flow direction. These secondary flows create velocity gradients within the fluid, causing particles or suspended entities to experience a drag force perpendicular to their motion.^53^ Dean flows (Fig. 1) are characterized by two counter-rotating vortices in the upper and lower halves of a microchannel. The intensity of these flows is quantified by a dimensionless parameter known as the Dean number ( $D_{e}$), which can be calculated as follows:^22,36^

$$D_{e}=R_{e}\sqrt{\frac{D_{H}}{2R}}=\frac{U_{F}D_{H}\varrho_{F}}{\mu_{F}}\sqrt{\frac{D_{H}}{2R}},$$ (7)

where R is the radius of curvature of the channel. In a straight channel, $D_{e}=0$. An increase in the channel cross section or flow rate will result in stronger Dean forces. For a given Dean number, the average Dean velocity ( $U_{Dean}$) is expressed as^36^

$$U_{Dean}=1.8\times 10^{-4}D_{e}^{1.63}.$$ (8)

Assuming Stokes drag, the Dean drag force ( $F_{D}$) on particles can be expressed as follows:^35^

$$F_{D}=3\pi \mu_{F}U_{Dean}a_{\;p}.$$ (9)

The focusing of particles to an equilibrium position in a curved microchannel depends on the ratio of the net inertial lift force to the Dean drag force, which must be greater than 0.04, making the Dean drag more dominant and enabling particle separation within a shorter channel length than in a straight channel with no Dean drag. However, if Dean drag is too dominant, particles will continue to move parallel to the flow direction, resulting in mixed flow rather than separation.^43^ The equilibrium positioning of particles in a curved microchannel with a rectangular cross section is dependent on the microchannel height (H) rather than the hydraulic diameter ( $D_{H}$) due to the variation in the shear rate across the channel cross section. Hence, the focusing criterion ( λ) is expressed as^35,43^

$$\lambda =\frac{a_{\;p}}{H}\geq 0.07.$$ (10)

## III. MATERIALS AND METHODS

### A. Device design

Figure 1 provides an illustration of our concept. The focusing criterion [Eq. (10)] defines the critical separation diameter in a spiral microchannel. The channel height (H) and width (W) of 70 and 200 μm are used for the spiral design, and the channel length is 90.33 mm. The radius of curvature of the spiral channel is 10 mm. The spiral channel is connected to a bifurcation inlet; the inner inlet (150 μm wide) of the bifurcation serves as the flow inlet for the sheath solution, while the outer inlet (50 μm wide) serves as the flow inlet for the sample suspension. Bifurcated outlets were used for the collection of bacteria and debris. The inner outlet (50 μm wide) is designed to collect large debris, while the outer outlet (150 μm wide) is designed to collect bacteria. Hou *et al.*^40^ shows that a smaller inner outlet is appropriate due to the narrower focusing of cells larger than the critical diameter near the inner wall of the channel.

### B. Device fabrication and characterization

The spiral channel is fabricated using soft-lithography in a clean-room environment. SU-8 2075, an epoxy-based photo-resist, is deposited onto a silicon wafer substrate and a two-step spin-coating process is performed with a spin coater (Laurell WS 650). In the first step of the spin-coating process, a spin speed of 500 rpm and an angular acceleration of 100 rpm/s is applied for 10 s. An additional 30 s of spinning is then conducted at a speed of 3000 rpm and acceleration of 300 rpm/s to ensure a uniform spread of the photo-resist on the silicon wafer substrate.

The spin-coated substrate is heated at $65{\;}^{{}^{\circ}}$C for 3 min and $95{\;}^{{}^{\circ}}$C for 9 min. Next, a UV exposure process is carried out using a mask aligner (ABM Mask Aligner) to transfer the spiral channel design on a photo-mask (Micro Lithography Services Ltd) onto the soft-baked substrate. The exposure energy and exposure time used during pattern transfer is 188 ${\mathrm{mJ}/\mathrm{cm}}^{2}$ and 19 s, respectively. A post-exposure bake (PEB) process is performed after UV exposure at $65{\;}^{{}^{\circ}}$C for 2 min and $95{\;}^{{}^{\circ}}$C for 7 min.

**FIG. 2.**
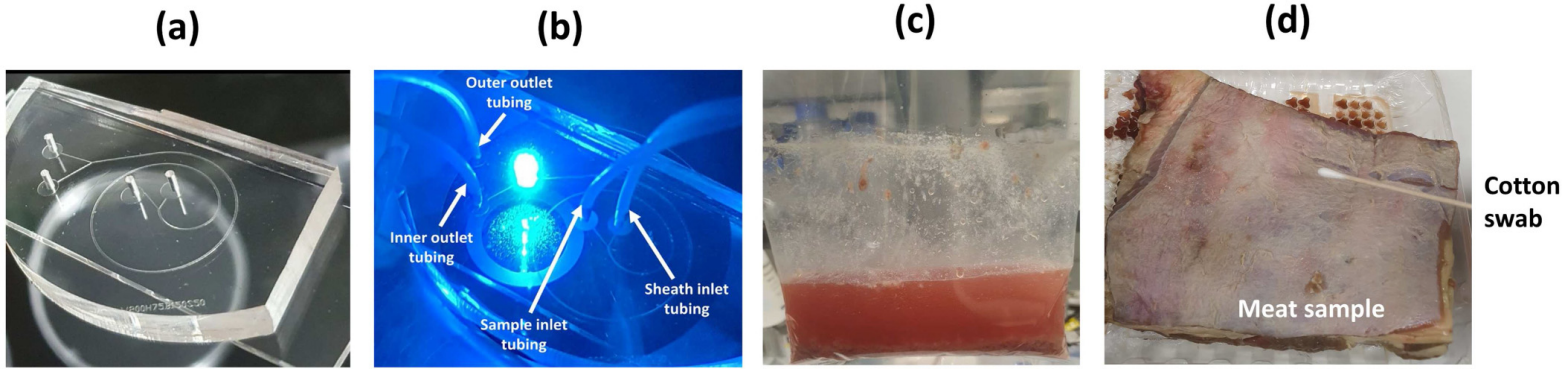
(a) Fabricated spiral microfluidic device. (b) Imaging experiment showing the tubing connection to the inner and outer inlets and outlets. (c) Ground meat sample suspension containing bacteria and non-bacterial debris. (d) Beef surface used for the preparation of meat swab suspension.

The development of the photo-resist is carried out by immersing the post-baked substrate in propylene glycol methyl ether acetate (PGMEA) solution for 7 min. The developed microchannel is characterized using an optical profilometer (Bruker Dektak XT Profilometer). A pre-designed acrylic mold is filled with sylgard 184 silicone elastomer base and silicone elastomer curing agent at a 10:1 mixing ratio to cast the developed spiral channel to polydimethylsiloxane (PDMS). The PDMS is cured at $65{\;}^{{}^{\circ}}$C for 120 min. The PDMS replica is bonded to a glass substrate [Fig. 2(a)] using oxygen plasma reactive ion etching (RIE) process at a power of 50 mW for 16 s at $25{\;}^{{}^{\circ}}$C. The microchannel is treated with Pluronic-F127 prior to experiments to reduce cell adhesion.

### C. Optimal flow rates for separation

To determine the optimal sample and sheath inlet flow rates for continuous separation, fluorescence imaging is employed. Two types of fluorescent yellow particles (Spherotech) with a mean size of 1.84 and 10.6 μm and a concentration of $2.92\times 10^{5}$ particles/ml and $1.53\times 10^{5}$ particles/ml, respectively, as well as fluorescent nile red particles (Spherotech) with a mean size of 6.04 μm and concentration of $8.26\times 10^{5}$ particles/ml are used. The 1.84 μm particle represented the average size of a bacterium while the 10.6 μm particle is used to represent larger particulates. The 6.04 μm particle is used to represent particulates closer to the separation threshold of the channel. The neMESYS Low Pressure module 290N flow pump (CETONI) is used to control the flow of suspensions and sheath solution (0.85% saline) into the spiral microchannel.

A Nikon Eclipse E600 inverted fluorescence microscope is used for the fluorescence imaging of the flow of the fluorescent particle suspension at the bifurcation outlet [Fig. 2(b)]. For the 1.84 and 10.6 μm fluorescent beads, a FITC (fluorescein isothiocyanate) filter with an excitation range between 465 and 495 nm is used, while for the 6.04 μm fluorescent beads, a Texas Red filter with an excitation range between 540 and 580 nm is used. A 10 × objective is used for capturing the flow images of 1.84 and 6.04 μm beads. A 20 × objective is used for the 10.6 μm beads.

Different sample-to-sheath flow rates are tested to determine the optimal flow rates. Two sample flow rates of 50 and 100 μl/min are tested in the imaging experiment and four different sample to sheath ratios of 1, 2, 4, and 8 are tested for each sample flow rate. These flow rate ranges are selected using Eq. (11), which estimates the minimum flow rate ( Q) required for particles above a critical diameter to focus at an equilibrium position near the inner wall of the microchannel. The minimum flow rate is expressed as^50^

$$Q=U_{F}WH=\frac{2\pi \mu_{F}WH^{3}}{3\rho_{F}L_{c}C_{L}a_{\;p}^{2}},$$ (11)

where $\mu_{F}$ is the dynamic fluid viscosity, $\rho_{F}$ is the fluid density, $L_{c}$ is the optimum channel focusing length, and $C_{L}$ is the lift coefficient, which varies from 0.05 to 0.02.^10^

### D. Bacteria suspension preparation

The first step in the sample preparation process is the preparation of an overnight culture of the selected bacterial strain. The overnight culture process is carried out by adding a single colony of bacteria to 10 ml of tryptic soy broth (TSB) and incubating overnight at $37{\;}^{{}^{\circ}}$C and 200 rpm. Next, a subculture is prepared by adding 0.5 ml of the overnight culture to 9.5 ml of TSB. For *Escherichia coli* (*E. coli*) MG1655 bacteria, 45 mins of incubation is sufficient to achieve an optical density (Absorbance at 600 nm, 1 cm path length) of approximately 0.5 while for *Staphylococcus aureus* (*S. aureus*) 6538 bacterial strain, an incubation time of 30 min is sufficient to achieve a mid-log bacteria growth phase. After each subculture, the optical density is measured using a spectrophotometer (Bichrom Libra S22). The subculture was subsequently centrifuged (Eppendorf 5702), and the supernatant (TSB) removed.

For live cell suspensions, the sub-culture is re-suspended into saline solution to make the stock suspension of live cells. The concentration of the stock suspensions is typically $∼10^{8}$ colony forming units (CFU)/ml. The dilution of the stock suspension to concentrations ranging between $∼10^{7}$ and $∼10^{2}$ CFU/ml is carried out using the appropriate volume of saline. In the case of meat swab preparation, buffered peptone water (Fort Richard Laboratories, Auckland NZ) is used instead of saline. Suspensions containing viable bacteria are subjected to tenfold dilution series in saline (0.85% (w/v) NaCl) and (0.1 ml) aliquots were spread onto tryptic soy agar (TSA) and incubated at 37 ${}^{{}^{\circ}}$C for 24 h. Plates with 20–200 colonies were counted to determine the CFU/ml of viable cells in the original sample.

### E. Separation experiments

#### 1. Live bacteria alone

To validate the recovery efficiency of live bacteria cells in the spiral microchannel using different bacteria strains and concentrations, suspensions of *E. coli* MG1655 and *S. aureus* 6538 were used. A concentration of $∼10^{5}$ CFU/ml for *E. coli* MG1655 and concentrations of $∼10^{5}$ and $∼10^{3}$ CFU/ml for *S. aureus* 6538 were used to perform the separation experiment. An ANOVA was applied to investigate if the bacteria species or concentration has a significant impact on the recovery at each outlet.

#### 2. Ground meat suspensions

A ground meat sample purchased from a local supermarket is used for the experiment. To prepare the sample, 25 g of ground meat sample is placed in a stomacher bag with 225 ml of peptone water. Thereafter, a stomacher (Seward Stomacher 400) is used for 60 s to agitate the meat sample in the peptone water [Fig. 2(c)]. Next, a cell strainer with a pore size of 40 μm is used to remove debris above 40 μm to prevent clogging of the microchannel. The sample syringe is filled with the suspension obtained from the cell strainer and the sheath syringe is filled with peptone water. The debris separation experiment is carried out using a sheath flow rate of 400 μl/min and a sample flow rate of 100 μl/min.

The percentage of debris recovery is determined through the measurement of the mass difference between the debris suspension collected after the cell strainer and the debris suspensions collected at both the inner and outer outlets. In this experiment, 5 ml of debris suspensions are collected from the inner outlet and outer outlet and centrifuged at 7000 × g for 15 min. Following centrifugation, the supernatant is carefully removed from each tube and the pellet is then frozen for 12 h ( −80 ${}^{{}^{\circ}}$C freezer room) and dried using a freeze drier (SanVac CoolSafe Freeze Drier) for a duration of 24 h. After the drying process, the mass of each tube is measured to validate the percentage of debris removed at the channel outlets.

Differential interference contrast (DIC) microscopy is performed using a Zeiss Axio-ImageM2 microscope and a 10 × objective for imaging the suspension collected after using the cell strainer and the debris suspension collected at the inner and outer outlet of the spiral microchannel. 5 μl of the respective suspensions are dispensed on a glass slide and covered with a coverslip for imaging. To improve the visualization and analysis of the size distribution of the DIC images recorded for the output suspensions, the image thresholding command is used in ImageJ.

Another experiment is carried out by spiking a ground meat suspension with *E. coli* MG1655 bacteria for a final concentration of $∼10^{5}$ CFU/ml. After the separation experiment is performed, the bacterial concentrations in the initial suspension, suspension after the cell strainer, and outer and inner outlet suspensions are determined using agar plate count method described in Sec. III D.

#### 3. Meat swabbing suspensions

The sample preparation is carried out using the meat swab suspension methodology recommended by the New Zealand Ministry for Primary Industries (MPI),^54^ which is used in New Zealand meat processing facilities.

Three wet and three dry cotton swabs were used to sample a 100 ${\mathrm{cm}}^{2}$ area of the meat surface [Fig. 2(d)]. These swabs were subsequently immersed in 14.85 ml of peptone water. Following this, the cotton swab shafts are snapped off aseptically against the inner wall of the peptone water bottle. The swab suspension is vortexed for 60 s to liberate bacteria and debris from the swab. The suspension is spiked with *E. coli* MG1655 by adding 0.15 ml of a $∼10^{5}$ CFU/ml suspension to the peptone water volume. This dilution results in a final bacterial concentration of $∼10^{3}$ CFU/ml. This suspension was subjected to the same separation procedure as the ground meat suspension: physical filtration through a cell strainer, and subsequent flow through a spiral microfluidic device using a sheath flow of 400 μl/min and suspension flow rate of 100 μl/min. Subsequently, a similar imaging procedure was followed to analyze debris size distribution and bacterial concentration.

The imaging analysis of the distribution of particulates in the suspension collected after the use of a cell strainer and the suspensions from the outer and inner outlets is carried out. For each suspension, 5 μl volume is dispensed onto a standard microscope glass slide, and a 20 × objective is used to acquire triplicate images.

## IV. RESULTS AND DISCUSSION

### A. Optimal flow rates for separation

Through our experimentation with various sheath-to-sample flow rates, we determined the optimal flow rates for both the sample and sheath fluids, resulting in continuous size-based particle separation for particles larger than the critical diameter. Specifically, we found that the ideal sample and sheath flow rates were 100 and 400 μl/min, respectively. At these optimal flow rates, the Reynolds number ( Re) and the Dean number ( De) within the channel are 79.4 and 5.7, respectively.

**FIG. 3.**
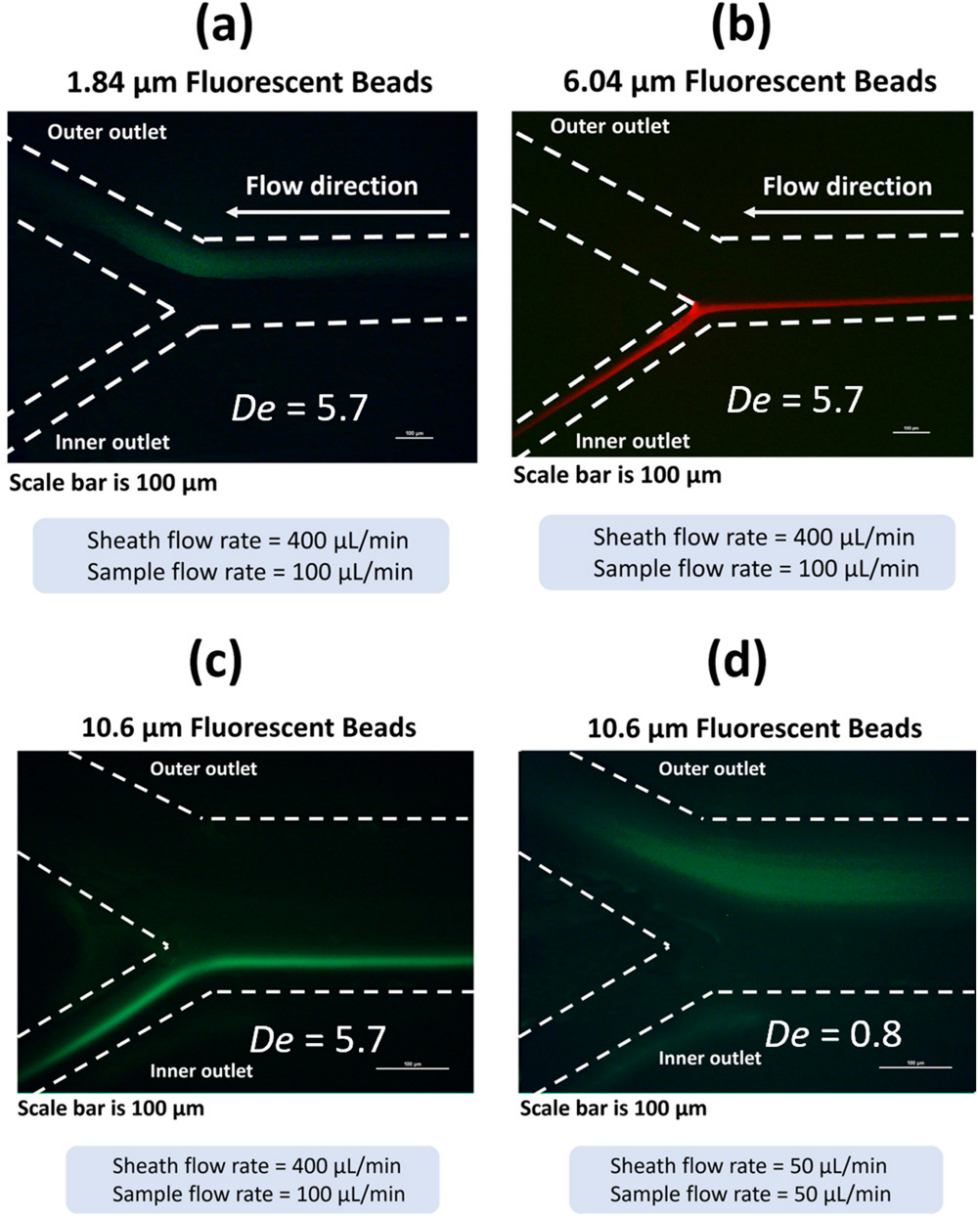
Focusing of the (a) 1.84 μm fluorescent particles to the outer outlet of the channel, (b) 6.04 μm fluorescent particles to the inner outlet of the channel, and (c) 10.6 μm fluorescent particles to the inner outlet of the channel for the optimal sample flow rate of 100 μl/min and sheath flow rate of 400 μl/min ( Re=79.4 and De=5.7). (d) The reduction in Dean number ( De) has a notable impact on the focusing behavior of the 10.6 μm fluorescent particles. By adjusting both the sample and sheath flow rates to 50 μl/min, the Dean number decreases to 0.8. This reduction in De leads to weaker Dean vortices within the microchannel, subsequently diminishing the influence of lift forces acting on the particles.

At a Dean number ( De) of 5.7, the 1.84 μm fluorescent particles, which have a size comparable to bacteria, experience a distinct behavior within the 70 μm high channel ( $a_{\;p}$/H ∼ 0.03) of the spiral microchannel [Fig. 3(a)]. These particles are observed to be focused near the outer wall of the spiral channel, aided by the presence of Dean vortices induced by the channel curvature. The outer outlet of the channel is designed wide enough to effectively collect these particles. In contrast, the 6.04 μm ( $a_{\;p}$/H ∼ 0.09) and 10.6 μm ( $a_{\;p}$/H ∼ 0.15) fluorescent particles exhibit a different behavior. They are observed to be focused near the inner wall of the channel [Figs. 3(b) and 3(c)]. This result demonstrates that at De=5.7, the lift force ( $F_{L}$) acting on the 6.04 and 10.6 μm particles near the inner wall of the channel is sufficient to focus them to equilibrium positions near the inner outlet wall.

As depicted in Fig. 3(d), a reduction in both the sheath and sample flow rates to 50 μl/min ( Re=11.3 and De=0.8) leads to a decrease in the magnitude of Dean vortices, consequently diminishing the influence of the lift force on particles exceeding the channel’s critical diameter. Notably, at De=0.8, the 10.6 μm particle ceases to be focused near the inner wall due to the lowered flow velocity, resulting in a reduced lift force effect. Instead, the 10.6 μm particle is observed to move toward the outer wall due to the Dean drag force ( $F_{D}$), surpassing the magnitude of lift force ( $F_{L}$). This is an indication that at a lower Dean number, the magnitude of Dean vortices decreases, resulting in weaker secondary flows. Consequently, the lift force acting on the particles is diminished. This reduced lift force influences the particle trajectory and equilibrium position within the channel, potentially altering the separation behavior.

**FIG. 4.**
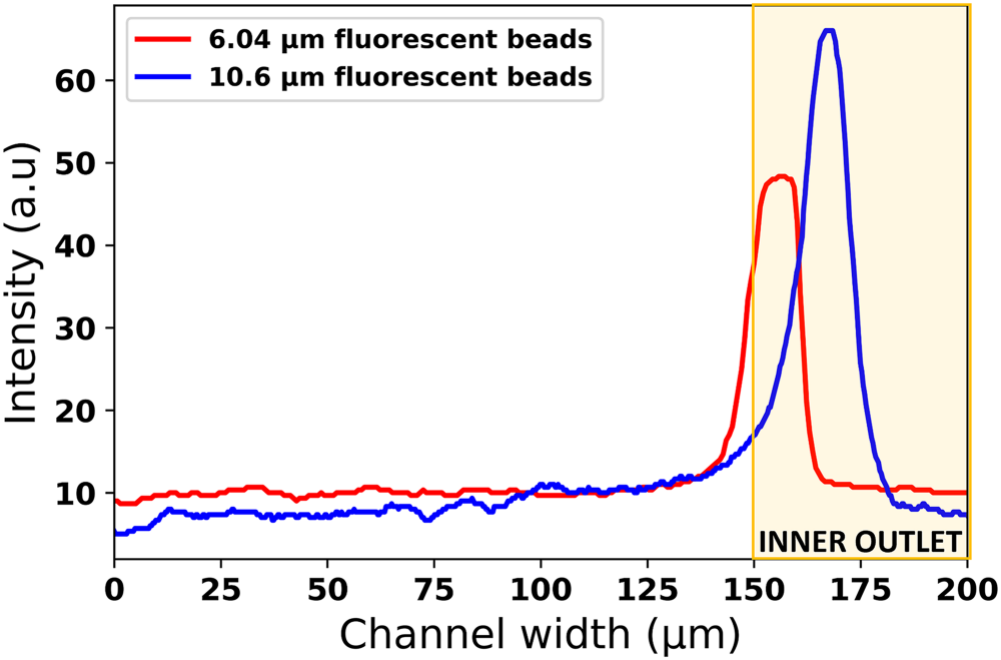
The intensity plots of the focused stream of the 6.04 and 10.6 μm fluorescent particles taken across the full channel width of 200 μm. The 6.04 μm beads focus in an equilibrium position near the inner wall of the channel but closer to the center of the bifurcation outlet, while the equilibrium position of the 10.6 μm beads is closer to the inner wall compared to the 6.04 μm beads.

We also observed the effect of particle size on the equilibrium position within the channel. As depicted in Fig. 4, the intensity plot provides a visual representation of the focusing width of the 6.04 and 10.6 μm particles near the inner wall of the channel. Specifically, the 6.04 μm particle converges toward an equilibrium position near the inner wall, positioned close to the center of the bifurcation outlet. In contrast, the 10.6 μm particle achieves a considerably closer equilibrium position to the inner wall. These observations suggest that as the particle size decreases while maintaining a constant Dean number, there is a progressive displacement of the equilibrium position away from the inner wall of the microchannel.

### B. Separation experiments

#### 1. Live bacteria alone

**FIG. 5.**
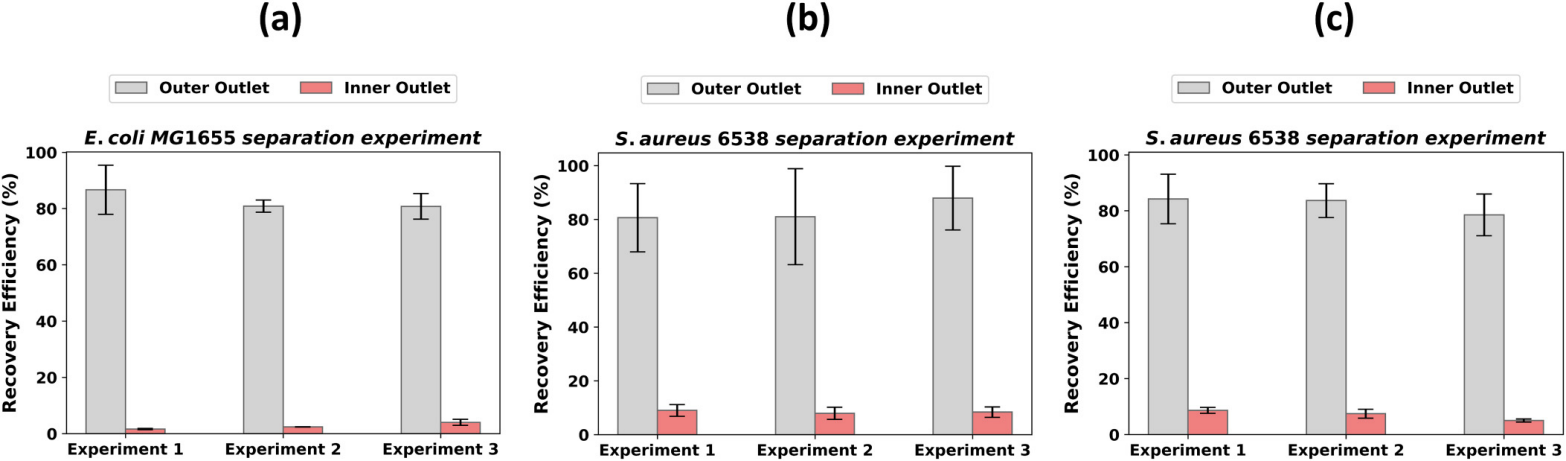
Recovery efficiencies at the outer and inner outlets for (a) $∼10^{5}$ CFU/ml *E. coli* MG1655, (b) $∼10^{5}$ CFU/ml *S. aureus* 6538, and (c) $∼10^{3}$ CFU/ml *S. aureus* 6538. Each bar represents the average of triplicate plate count measurements, and the error bar represents the standard deviation of the triplicate plate count.

To better understand recovery efficiencies, two different species and shapes of bacteria, *E. coli* MG1655 (rod shaped) and *S. aureus* 6538 (spherical), were investigated. Further, the recovery efficiency associated with two different total concentrations of *S. aureus* were examined. Recovery efficiencies exceeding 80% were observed for *E. coli* MG1655 at $∼10^{5}$ CFU/ml. A small proportion of cells did exit the inner outlet of the channel [Fig. 5(a)]. An average ± standard deviation of 82.8 ± 5.2% and 2.6 ± 0.5% of bacteria were collected at the outer and inner outlet, respectively.

Recovery efficiencies exceeded 78% at the outer outlet for $∼10^{5}$ CFU/ml *S. aureus* 6538 and averaged 82.1 ± 7.5% across three experiments [Fig. 5(b)]. At the inner outlet an average of 7.0 ± 1.4% of cells were collected. A possible explanation for a greater proportion of cell recovery at the inner outlet for *S. aureus* compared to *E. coli* could be due to the inherent ability of *S. aureus* 6538 to form multiple cell clusters, which can result in an increase in total particulate size above the separation threshold. The recovery efficiency at the outer outlet was not significantly different between the two species of bacteria (p-value > 0.05), and was significantly different at the inner outlet (p-value < 0.05).

Reducing bacteria concentration of *S. aureus* 6538 to $∼10^{3}$ CFU/ml has minimal impact on the recovery efficiency [Fig. 5(c)]. The average recovery percentage over the three experiments for the $∼10^{3}$ CFU/ml concentration are 83.2 ± 14.1% (outer outlet) and 8.4 ± 2.1% (inner outlet) respectively. Recovery efficiencies at both the inner and outer outlets were not significantly different with the different concentrations investigated (p-value > 0.05).

Although determining the precise size distribution of bacteria in the sample mixture is challenging, it is plausible that some bacteria may have dimensions below 1 μm. Drawing insights from the investigation conducted by Johnston *et al.*,^55^ which centered on evaluating the application of Dean flow separation for particles ranging from 1 to 3 μm, utilizing a channel width and height of 20 μm, it was observed that effective focusing of 1 μm microspheres proved unachievable due to the excessive pressures required for optimal focusing conditions. In our separation method, it is noteworthy to emphasize that bacteria cells below 1 μm in size may exhibit divergent behavior compared to the study by Johnston *et al.*^55^ This discrepancy arises from the fact that their study focused on establishing a critical diameter in the vicinity of 1 μm, intending to utilize lift forces for concentrating particles near the inner walls of the microchannel. However, this approach proved ineffective due to the presence of weak Dean vortices. In our approach, the aim is not to achieve focusing of particles below 1 μm near the inner wall of the channel; instead, our objective is to induce re-circulation of these particles within the Dean vortices and exploit a strong Dean drag force to migrate them toward the outer walls of the channel.

The total recovery rates observed from both channel outlets indicate that there is cell loss. One possible explanation for this observation could be the adhesion of cells to the channel’s surface or the occurrence of cell death during the flow experiments. During the flow experiments, cells might adhere to the surface of the channel, preventing their detachment and subsequent loss. This adhesion can be attributed to various factors, such as the hydrophilic properties of the glass material used for bonding the microchannel. Further investigation into the nature of possible adhesion and its impact on cell recovery would provide valuable insights into the experimental setup. Another possible factor that could account for cell loss is the possibility of cell death during the flow experiments. The conditions within the channel, including fluid shear stress may lead to cell stress and ultimately cell death. However, a study carried out by Lange *et al.*^56^ discovered that the shear stress tolerance of different microorganisms varies and can be attributed to disparities in the tensile strength of respective cell walls or membranes. In the case of *E. coli*, which has a thin peptidoglycan layer, the shear stress tolerance threshold is 1250 Pa.

The maximum shear rate in the spiral channel was estimated to be approximately 40 000 $s^{-1}$. At the optimal flow conditions, the maximum shear stress in the spiral microchannel was estimated to be 40 Pa. This indicates that the optimal flow rates and the design parameters of the spiral channel result in a shear stress below the threshold that would affect the viability of *E. coli* bacteria. We infer that the channel geometry and flow conditions employed in our study would have minimal detrimental effects on the viability of bacterial cells.

#### 2. Bacterial from ground meat suspensions

**FIG. 6.**
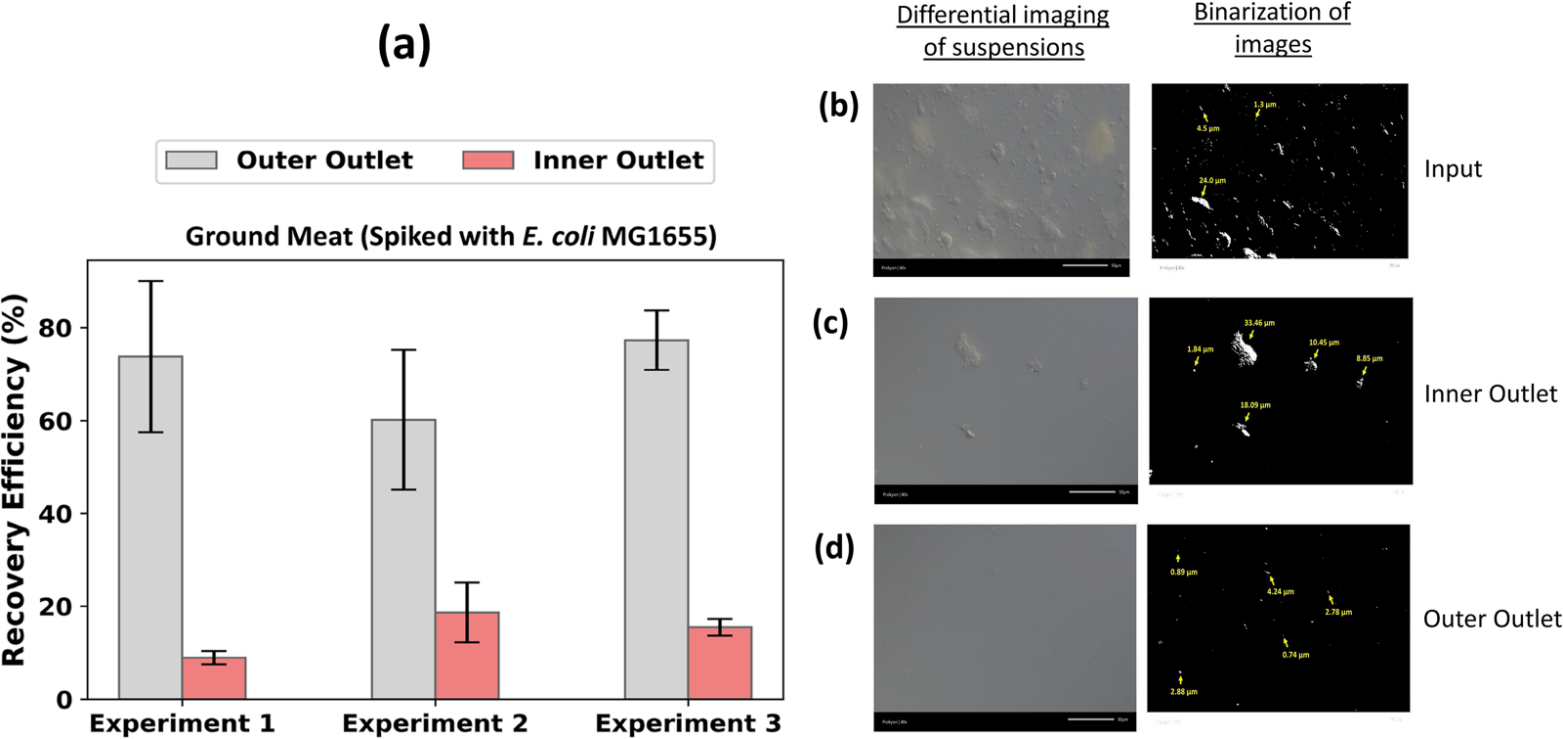
(a) The recovery efficiency of *E. coli* MG1655 bacteria from ground meat debris spiked with $∼10^{5}$ CFU/ml. Each bar represents the average of triplicate plate count measurements, and the error bar represents the standard deviation of the triplicate plate count. Size distribution of (b) the input suspension, which is a mixture of both debris and bacteria, (c) debris in the suspension collected at the inner outlet showing particulate sizes above the critical diameter of the channel, and (d) particulates in the suspension collected at the outer outlet of the microchannel. The binarization of the image is presented for better visualization and size measurements. The scale bars are 50 μm.

The recovery efficiency of *E. coli* MG1655 bacteria was reduced in the presence of ground meat debris relative to bacteria alone. The average recovery of *E. coli* was 70.6 ± 19.5% and 17.1 ± 4.1% at the outer and inner outlets, respectively, for ground meat suspensions [Fig. 6(a)]. The potential cause of the increased percentage of bacteria at the debris outlet is the possible attachment of bacteria cells to the large debris.

We determined that the average percentage of debris collected at the inner outlet is 49.4% while 43.4% of debris is removed at the outer outlet. As a result of the non-homogeneous nature of the debris, complete removal of debris from the bacteria sample may not be possible if a large percentage of the debris are below the critical diameter ( ∼5 μm). Particles small than the ∼5 μm critical diameter were present in the ground meat suspension after the cell strainer step [Fig. 6(b)]. This imaging result validates that the particulates in a suspension of debris extracted from meat samples are non-homogeneous in nature, which is in agreement with the findings of Clime *et al.*^45^ The analysis of the debris size and shape in the suspension collected at the inner outlet in Fig. 6(c) shows debris with sizes above the critical diameter/separation threshold of ∼5 μm. This result indicates that inertial lateral migration of debris of different shapes and sizes above the critical diameter can be achieved. Figure 6(d) shows that the average sizes of the debris present are less than the critical diameter of ∼5 μm. The debris present in the outer outlet are bacteria-sized, indicating that debris above the critical diameter of the channel are effectively separated.

#### 3. Bacterial from meat swab suspensions

**FIG. 7.**
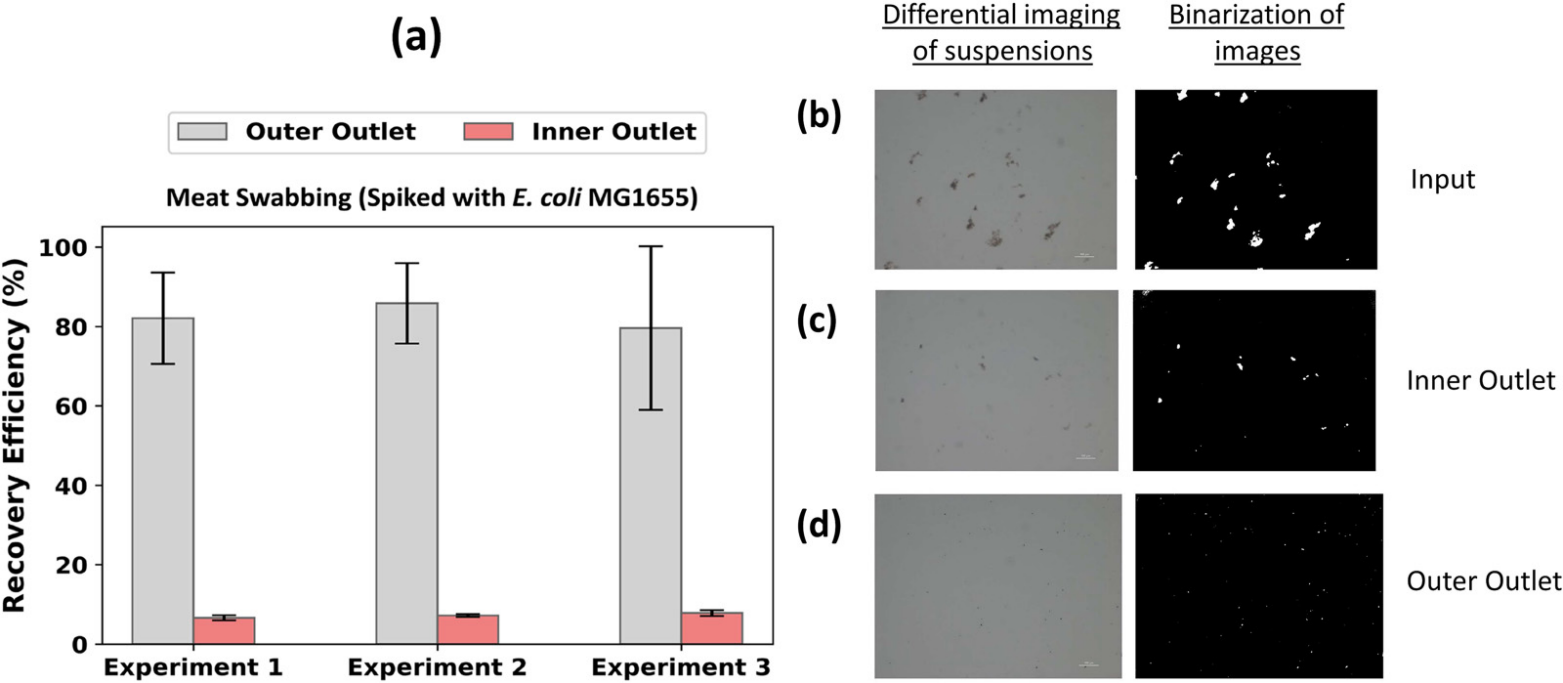
(a) The recovery efficiency of *E. coli* MG1655 bacteria from debris in a meat swabbing suspension spiked with $∼10^{3}$ CFU/ml concentration. Each bar represents the average of triplicate plate count measurements, and the error bar represents the standard deviation of the triplicate plate count. (b) Distribution of particle sizes in the input suspension, which consists of both debris and bacteria. (c) Particulate sizes above the critical channel diameter were observed in the size distribution of debris in the suspension collected at the inner outlet. (d) The size distribution of particulates in the suspension collected at the microchannel’s outer outlet. The scale bars are 100 μm.

The separation of bacteria cells from debris in a meat swab suspension shows an average recovery of 82.5 ± 14.1% and 7.2 ± 0.6% at the inner and outer outlets, respectively [Fig. 7(a)]. This result shows the effective separation of bacteria from a non-homogeneous mixture of debris in the meat swabbing suspensions. In this case, the recovery efficiency is ∼10% greater than the average efficiency for ground meat suspension.

Analysis of the particulates of meat swab suspensions after filtering through cell strainers indicated a range of particulate sizes [Fig. 7(b)]. This debris mixture consists of cotton fiber, fascia, and muscle tissue with different shapes and sizes. Figure 7(c) indicates that debris larger than the critical diameter of ∼5μm are present. Because the suspension collected at the inner outlet has been diluted by the sheath flow during the experiment, the inner outlet image is more diluted than that of the after cell strainer. Figure 7(d) shows the size distribution of particulates in the outer outlet of the microchannel of which many are smaller than the critical diameter. There is a possibility that the particulates at the outer outlet of the channel are a combination of bacterial cells and debris that are smaller than the critical diameter ( ∼5μm) of the microchannel. This result validates that a non-homogeneous mixture of debris above the critical diameter of separation can be effectively collected at the inner outlet of the channel and both bacteria and bacteria-sized debris can be collected at the outer outlet of the channel.

## V. CONCLUSION

We demonstrated that inertial microfluidics can be successfully applied for separating bacterial cells from debris of meat swab suspensions at a Dean number of 5.7. An average recovery efficiency of over 80% is achieved for both *E. coli* and *S. aureus* bacteria for non-debris samples at the outer outlet of the spiral channel. However, the average percentage of cells recovered in the inner outlet of the channel increased from *E. coli* MG1655 to *S. aureus* 6538. We found that the average recovery efficiency of *E. coli* from meat swab suspensions was 82.5%, higher than that of whole ground meat. Additionally, only approximately half of the ground meat debris has been removed from the bacterial cells. The complete removal of all debris in the sample suspension cannot be achieved because of the non-homogeneous nature of the debris, which results in sizes below the critical diameter of the separation channel.

There is a potential to use this separation methodology for sample preparation in areas, such as food safety inspection and environmental samples (e.g., drinking water supplies). Bacteria separated from debris can be analyzed in detail. Optical methods can be used to interrogate the bacteria in a sample using fluorescent stains or fluorescent tags, e.g., PI/SYTO9 to evaluate viability based on membrane integrity^57^ or fluorescent antibody tags to enumerate particular species.^58^

For future research, it will be useful to experimentally determine if the percentage of cell loss is the result of cell death or cell adhesion to the channel surface. A viability test is a potential option for assessing cell loss to assess for the presence of dead cells in the recovered sample. To minimize cell adhesion to the channel, future research can explore the use of surfactants or bacteria growth media that can reduce the adhesion of cells. As part of the sample preparation process, a 40 μm cell strainer was used to reduce the size distribution of the debris suspension. By integrating on-chip filters into the separation microchannel, this extra manual experimental step may be automated to save time.

## Data Availability

The datasets supporting the conclusions of this paper will be made available to readers.
